# Particular Aspects of Cardiac Rhythm Disorders in Symptomatic Children and Adolescents

**DOI:** 10.3390/children13081089

**Published:** 2026-08-17

**Authors:** Georgiana Bianca Constantin, Iuliana Moraru, Cristina Șerban, Mădălin Guliciuc, Raul Mihailov, Bogdan Ioan Ștefănescu

**Affiliations:** 1 Morphological and Functional Sciences Department, Faculty of Medicine and Pharmacy, Research Center in the Medical-Pharmaceutical Field, “Dunarea de Jos” University of Galati, 800010 Galati, Romania; 2 Clinical Medical Department, Faculty of Medicine and Pharmacy, “Dunarea de Jos” University of Galati, 800010 Galati, Romania; 3 Clinical Surgical Department, Faculty of Medicine and Pharmacy, “Dunarea de Jos” University of Galati, 800010 Galati, Romania; cristina.serban@ugal.ro (C.Ș.); madalin.guliciuc@ugal.ro (M.G.); raul.mihailov@ugal.ro (R.M.); bogdan.stefanescu@ugal.ro (B.I.Ș.)

**Keywords:** cardiac rhythm disorders, symptomatic, children

## Abstract

**Background**: Cardiac arrhythmias in children and adolescents may present with nonspecific symptoms such as precordial pain, palpitations, and syncope. We evaluated the clinical characteristics and rhythm findings of symptomatic pediatric patients and examined cross-sectional associations between reported symptoms and selected rhythm diagnoses, with particular attention to the diagnostic and management yield of ambulatory Holter monitoring. Because symptom timing is not always captured during diagnostic monitoring, associations between reported symptoms and detected rhythm abnormalities must be distinguished from temporal symptom–rhythm correlation and causation. **Methods**: We conducted an observational study of 119 children and adolescents aged 1–18 years, who were evaluated at a tertiary pediatric hospital for symptoms potentially suggestive of cardiac rhythm abnormalities. Data included demographic characteristics, clinical symptoms, personal and family history, resting electrocardiography, ambulatory Holter monitoring, and exercise testing where clinically indicated. The primary analysis was cross-sectional. Because symptom timing during ambulatory monitoring was not recorded, the study was not designed to establish temporal symptom–rhythm correlation or causality. Descriptive analyses were performed for the final 119-participant cohort. Selected symptom–rhythm associations were evaluated using the contingency table methods with odds ratios (ORs), 95% confidence intervals (CIs), and Fisher’s exact tests where appropriate. Holm’s adjustment was applied to the prespecified family of reconstructed symptom–rhythm comparisons. **Results** were interpreted according to both statistical evidence and effect-size precision. Results: The cohort comprised 119 participants, including 75 females (63.0%) and 44 males (37.0%). The most frequently reported symptoms were precordial pain (105/119, 88.2%), palpitations (80/119, 67.2%), and syncope or lipothymia (39/119, 32.8%). A family history of sudden cardiac death was reported by 28 participants (23.5%). Forty-two participants (35.3%) had a normal resting ECG, while 77 (64.7%) underwent ambulatory Holter monitoring. Among those undergoing Holter monitoring, 65/77 (84.4%) had rhythm abnormalities detected exclusively by ambulatory monitoring and not identified on the resting ECG. Holter findings resulted in at least one documented management change in 38/77 participants (49.4%). Management categories were not mutually exclusive and included lifestyle or activity adjustment in 21 participants, initiation of antiarrhythmic therapy in 19, medication monitoring in 19, and targeted cardiac imaging, including cardiac magnetic resonance imaging, in 5. In this cross-sectional analysis, reported syncope/lipothymia was strongly associated with the presence of complete atrioventricular block (10/39 [25.6%] versus 1/80 [1.3%]; OR 27.24, 95% CI 3.34–222.29; Fisher’s exact *p* < 0.001; Holm’s adjusted *p* approximately 0.0004). The unadjusted association between syncope/lipothymia and the WPW-labelled diagnosis did not remain statistically significant after Holm’s adjustment. No statistically significant associations were identified between syncope/lipothymia and PSVT or VT. **Conclusions**: In this pediatric cohort, ambulatory Holter monitoring identified rhythm abnormalities not detected on resting ECG in a substantial proportion of monitored participants and resulted in documented changes in clinical management in approximately half of those monitored. Reported syncope/lipothymia was strongly associated with the presence of complete atrioventricular block in the reconstructed cross-sectional analysis. Because symptom timing during monitoring was not recorded, these findings do not establish that the detected rhythm abnormalities caused the reported symptoms or that symptoms predict future rhythm outcomes. Further studies using prospectively defined symptom–rhythm event recording and longitudinal follow-up are needed to evaluate temporal correlation and prognosis.

## 1. Introduction

The incidence of cardiac rhythm disorders in symptomatic children is approximately 55.1 per 100,000 emergency department presentations. In general, the number of symptomatic patients represents between 30% and 45% of those ultimately diagnosed with a form of arrhythmia, although the majority of arrhythmias in this age group may be asymptomatic [1]. Cardiac rhythm abnormalities in children and adolescents encompass a broad spectrum of conditions, ranging from benign ectopy and physiological rhythm variation to clinically significant tachyarrhythmias, bradyarrhythmias, and conduction disorders. Although many rhythm abnormalities in this age group are asymptomatic or well tolerated, symptoms such as palpitations, precordial pain, dizziness, fatigue, dyspnea, and syncope may prompt cardiac evaluation. A normal clinical examination or resting electrocardiogram does not necessarily exclude an intermittent rhythm abnormality, particularly when the suspected event is episodic [1].

Medical emergencies caused by cardiac rhythm disorders in pediatrics are relatively rare because these conditions are generally well tolerated in this age group. However, in many cases, the first manifestation of a cardiac rhythm disorder may be sudden cardiac death. Even among the symptomatic patients, who have experienced episodes of palpitations, precordial pain, and syncope, the clinical examination may be normal [2,3].

The incidence of paroxysmal supraventricular tachycardia (PSVT) varies in the literature from 1 in 250,000 to 1 in 250 cases, and among these, approximately 25% of PSVT cases are ultimately diagnosed with Wolff–Parkinson–White (WPW) syndrome [4]. Given that the incidence of sudden death among children with WPW syndrome and syncopal episodes is 0.5%, and that these patients have an indication for ablation, their identification and monitoring are extremely important [5]. Wolff–Parkinson–White terminology requires diagnostic precision. Ventricular pre-excitation is an electrocardiographic finding, whereas WPW syndrome generally refers to the clinical context of pre-excitation associated with documented tachyarrhythmia or relevant clinical manifestations. These concepts should not be treated as interchangeable.

Sillca MB emphasized that the incidence of ventricular extrasystoles (VEs) was identified in 0.8–2.2% of children on a standard electrocardiogram, whereas 24-h Holter ECG monitoring revealed a significantly higher prevalence, reaching 18% in newborns and 50% in adolescents [6].

Regarding bradyarrhythmias, sick sinus syndrome has a low incidence in the pediatric age group, and most affected patients are asymptomatic. Furthermore, data from the literature report an incidence of atrioventricular blocks ranging from 1 in 15,000 to 1 in 25,000 cases [7]. John M. highlights the importance of medical history, particularly regarding medication and diet, given that, for example, the use of nasal decongestants may trigger cardiac arrhythmias [3].

In recent years, on the one hand due to increased vigilance, and on the other hand due to the development of new non-invasive and invasive investigative methods, the incidence of diagnosing cardiac rhythm disorders in the pediatric age group has increased considerably. A study conducted in India and published in 2016 aimed to identify and analyze the characteristics of potentially lethal cardiac arrhythmias in children. The study included subjects ranging from infancy to 12 years of age, excluding those with benign arrhythmias such as sinus tachycardia, sinus arrhythmia, and sinus bradycardia. In total, the study cohort consisted of 60 patients. The study showed that ventricular extrasystoles had the highest incidence, followed by sick sinus syndrome and supraventricular tachycardia. It also found an association between WPW syndrome and cardiac abnormalities that had not been identified prior to inclusion in the study. The conclusion of this study was that cardiac rhythm disorders in children may occur at any point in life, and that a high index of suspicion is necessary for their identification, since most are asymptomatic, often until an episode of sudden death occurs [8].

The classification of arrhythmias associated with risk of sudden cardiac death was based on the clinical and electrocardiographic characteristics of the arrhythmias observed in our cohort and on contemporary guideline and consensus recommendations concerning arrhythmias and substrates associated with increased risk of malignant ventricular arrhythmia or sudden cardiac death. According to the recent guidelines, we consider arrhythmias that directly represent malignant ventricular arrhythmia (e.g., sustained ventricular tachycardia or ventricular fibrillation) and arrhythmias or substrates that may confer risk only in specific clinical contexts (e.g., ventricular pre-excitation, certain inherited electrical disorders, or selected high-risk ventricular ectopy) [2].

A normal clinical examination or resting electrocardiogram does not necessarily exclude an intermittent rhythm abnormality, particularly when the suspected event is episodic. The evaluation of symptomatic children may therefore require a combination of clinical history, physical examination, standard electrocardiography, ambulatory monitoring, exercise testing, and, when clinically indicated, cardiac imaging or other specialized investigations. The diagnostic yield of these investigations depends on the frequency and duration of the suspected rhythm abnormality and on the timing of monitoring relative to symptoms. Ambulatory monitoring may be particularly useful when the resting ECG is normal, but symptoms are intermittent. Holter monitoring can identify rhythm abnormalities that are not present during a short resting ECG recording and may lead to changes in treatment, activity recommendations, medication management, or further diagnostic evaluation.

The interpretation of symptom–rhythm relationships requires particular caution. A patient may report palpitations, chest discomfort, or syncope and may also be found to have a rhythm abnormality during a subsequent evaluation. However, unless the symptom occurs during a documented rhythm event, the presence of both findings does not establish temporal correlation or causality. Similarly, a cross-sectional association cannot establish whether a symptom is a prognostic factor or that it predicts future development of a specific arrhythmia.

The present study evaluated children and adolescents presenting for assessment of symptoms potentially suggestive of cardiac rhythm abnormalities. The objectives were to describe the clinical and rhythm findings in our 119-participant cohort, to examine selected cross-sectional associations between reported symptoms and documented rhythm diagnoses, and to quantify the diagnostic and management impact of ambulatory Holter monitoring. The study was not designed to establish longitudinal prognostic risk. We also aimed to establish an investigation protocol in order to optimize the diagnosis of arrhythmias.

## 2. Materials and Methods

### 2.1. Study Design and Setting

This was an observational study of children and adolescents evaluated at a tertiary pediatric hospital for symptoms potentially suggestive of cardiac rhythm abnormalities.

The primary analysis was cross-sectional. Clinical symptoms were obtained from the medical history and questionnaire, while rhythm diagnoses were established through the clinical evaluation and investigations performed as clinically indicated. The study did not include a prospectively predefined longitudinal follow-up schedule for the primary symptom–rhythm analysis. Any subsequent clinical reassessments reflected routine clinical practice and were not performed according to a predefined research follow-up protocol. Therefore, follow-up information is reported descriptively.

All consecutive patients meeting the eligibility criteria during the study period were invited to participate. Written informed consent was obtained from the patients’ legal representatives. The study was conducted in accordance with applicable ethical principles and the Declaration of Helsinki and was approved by the Ethics Committee of the Saint John Emergency Clinical Hospital in Galați.

### 2.2. Study Population

The final study cohort comprised 119 children and adolescents aged 1–18 years who were evaluated between January 2021 and December 2025. This was an observational cross-sectional study based on the clinical assessment performed at the time of inclusion. Although some participants subsequently attended routine clinical reassessment according to standard medical care, no prospective follow-up schedule formed part of the study design.

The study was conducted following the provision of written informed consent by the patients’ legal representatives. The consent process was conducted in accordance with applicable national legislation and internationally recognized ethical principles governing research involving human subjects, including the relevant regulations of the European Union and the principles of the Declaration of Helsinki. The legal representatives were also informed that, following completion of the clinical evaluation and required investigations, temporary or permanent restriction of physical activity might be recommended if the patient was found to have a cardiac rhythm disorder associated with a potential risk of sudden cardiac death.

The individual questionnaire was used as a working instrument that represented a requirement for all patients enrolled in this study group (or their legal representatives) to complete comprehensive questionnaires, prepared in accordance with the current legislation of the World Health Organization and the European Union regarding research on human subjects in the medical field. These included complete information such as date of birth, perinatal history, physiological and pathological personal history, family history, lifestyle, dietary habits, personal habits or habits associated with family members, the presence or absence of cardiac-related symptoms (including episodes of palpitations, precordial pain, dyspnea, fatigue, and lipothymia), the number of hours spent in front of the television or other devices (tablet, phone, video games), as well as information regarding the use of vitamins, immunostimulants, or anabolic substances. The standard duration for completing the questionnaires was approximately 15 min.

The questionnaire and clinical assessment recorded demographic characteristics, perinatal history, personal medical history, family history, lifestyle factors, and cardiac-related symptoms, including palpitations, precordial pain, dyspnea, fatigue, dizziness, and syncope or lipothymia.

Patients were excluded when they or their legal representatives declined participation, when congenital heart malformations were present, when a previously established rhythm disorder was already known before referral, when there was a history of cardiac surgery, or when a known neurological disorder or disability could substantially affect the interpretation of the presenting symptoms.

### 2.3. Clinical and Diagnostic Evaluation

The clinical evaluation included medical history, physical examination, resting electrocardiography, and additional investigations according to the presenting symptoms and clinical findings.

Ambulatory Holter monitoring was performed in 77 of the 119 participants. The remaining participants were evaluated using the available clinical and electrocardiographic investigations without Holter monitoring.

The final cohort included:

- 42/119 participants (35.3%) with a normal resting ECG;
- 77/119 participants (64.7%) who underwent Holter monitoring;
- 65/77 participants (84.4%) in whom rhythm abnormalities were detected exclusively by Holter monitoring and were not identified on the resting ECG.

Exercise testing and other investigations were performed when clinically indicated.

### 2.4. Holter Monitoring

The diagnostic yield of ambulatory monitoring was defined as the proportion of monitored participants in whom a rhythm abnormality was detected exclusively by Holter monitoring and was not identified on the resting ECG.

Management impact was defined as the occurrence of at least one documented clinical management change attributable to the Holter findings.

Among the 77 participants who underwent Holter monitoring, 38 (49.4%) had at least one documented management change. Management categories were not mutually exclusive because individual participants could experience more than one intervention.

The documented categories of management change included:

- initiation of antiarrhythmic therapy;
- medication monitoring;
- medication dosage adjustment where applicable;
- lifestyle or activity modification, including activity restrictions where clinically indicated;
- targeted cardiac imaging, including cardiac magnetic resonance imaging.

The number of participants with each management category was recorded separately. Because individual participants could have more than one type of intervention, the sum of category-specific interventions could exceed the number of participants with any management change.

### 2.5. Classification of Rhythm Abnormalities

Rhythm abnormalities were classified according to the documented electrocardiographic or ambulatory findings.

The study distinguishes, where the available data permit, between:

1. an electrocardiographic ventricular pre-excitation pattern;
2. documented tachyarrhythmia;
3. a clinically defined WPW syndrome.

Where the archived aggregate data did not allow these categories to be independently separated, the more conservative term “WPW-labelled diagnosis” was used.

Similarly, ventricular tachycardia, premature ventricular contractions or ventricular ectopy, sinus node dysfunction, tachy–brady syndrome, second-degree atrioventricular block, and complete atrioventricular block were reported according to the diagnostic category available in the study records. No additional mechanistic or severity classification was inferred when it could not be independently verified from the available data.

The classification of rhythm abnormalities with potential clinical relevance was informed by the documented rhythm diagnosis and contemporary clinical recommendations. However, the presence of a rhythm diagnosis in this cross-sectional cohort was not interpreted as equivalent to an established future risk of sudden cardiac death.

### 2.6. Statistical Analysis

The final analytic cohort consisted of 119 participants. Descriptive statistics were used to summarize demographic characteristics, clinical symptoms, rhythm findings, resting ECG results, Holter monitoring, and management changes. Categorical variables are presented as counts and percentages, while continuous variables are summarized using appropriate measures of central tendency and dispersion.

Odds ratios (ORs), 95% confidence intervals (CIs), and Fisher’s exact tests were calculated from reconstructed 2 × 2 contingency tables derived from the cleaned final dataset. These reconstructed tables corresponded to the prespecified symptom–rhythm comparisons included in the analysis. Holm’s adjustment was subsequently applied to this family of comparisons to account for multiple testing.

The prespecified symptom–rhythm comparisons included:

- precordial pain versus WPW-labelled diagnosis;
- precordial pain versus PSVT;
- precordial pain versus ventricular tachycardia;
- precordial pain versus complete atrioventricular block;
- syncope/lipothymia versus WPW-labelled diagnosis;
- syncope/lipothymia versus PSVT;
- syncope/lipothymia versus ventricular tachycardia;
- syncope/lipothymia versus complete atrioventricular block.

Spearman’s rank correlation coefficient was used to evaluate associations involving ordinal or binary variables presented in the correlation analyses. An independent-samples *t*-test was used to compare groups where appropriate. Simple linear regression was performed to explore the relationship between blood pressure and the number of reported syncopal episodes.

Because symptom timing during ambulatory monitoring was not recorded, no statistical analysis was interpreted as demonstrating temporal symptom–rhythm correlation or causality. All statistical tests were two-sided, with *p* < 0.05 considered statistically significant. Analyses were performed using IBM SPSS Statistics version 24 and Microsoft Excel 2017.

## 3. Results

### 3.1. Participant Characteristics

The study included 119 children and adolescents with a mean age of 10.92 years. Most participants lived in urban areas (82/119, 68.9%), while 37 (31.1%) lived in rural areas. The cohort included 75 females (63.0%) and 44 males (37.0%).

Perinatal history was unremarkable in 68 participants (57.1%). The most frequently reported perinatal conditions were neonatal jaundice in 33 participants (27.7%) and respiratory distress in 11 (9.2%); both conditions were reported in 7 participants (5.9%).

### 3.2. Presenting Symptoms

The distribution of presenting symptoms is shown in Table 1. Precordial pain was the most frequently reported symptom. Overall, 105/119 participants (88.2%) reported precordial pain, including 95 (79.8%) who reported persistent or frequent symptoms and 10 (8.4%) who reported occasional episodes.

Palpitations were reported by 80/119 participants (67.2%), including 66 (55.5%) who reported frequent symptoms and 14 (11.8%) who reported occasional symptoms. Lipothymia or syncope was reported by 39 participants (32.8%). Vertigo was reported by 25 participants (21.0%), including 21 (17.6%) with persistent symptoms and 4 (3.4%) with occasional symptoms.

Dyspnea was reported with varying descriptions and degrees of exertional association. Because the original symptom categories were not mutually standardized, the detailed distribution is presented in Table 1.

### 3.3. Rhythm Findings in Relation to Precordial Pain

The presence of precordial pain was evaluated in relation to documented rhythm diagnoses. Because the study did not record whether symptoms occurred simultaneously with a documented rhythm event, these analyses represent cross-sectional associations and do not establish temporal symptom–rhythm correlation or causality.

Among the 10 participants who reported occasional precordial pain, the documented rhythm findings included atrial extrasystoles in 5 (4.2% of the total cohort), including systematized atrial extrasystoles in 1 (0.8%); ventricular extrasystoles in 4 (3.4%), including systematized ventricular extrasystoles in 2 (1.7%); sinus tachycardia in 6 (5.0%); a WPW-labelled diagnosis in 3 (2.5%); PSVT in 1 (0.8%); tachy–brady syndrome in 3 (2.5%); sick sinus syndrome in 1 (0.8%); second-degree atrioventricular block in 1 (0.8%); and complete atrioventricular block in 1 (0.8%).

Among participants who did not report precordial pain, the documented findings included atrial extrasystoles in 4 (3.4%), including systematized atrial extrasystoles in 2 (1.7%); ventricular extrasystoles in 4 (3.4%), including systematized ventricular extrasystoles in 1 (0.8%); sinus tachycardia in 11 (9.2%); PSVT in 2 (1.7%); tachy–brady syndrome in 3 (2.5%); ventricular tachycardia in 2 (1.7%); sick sinus syndrome in 1 (0.8%); a WPW-labelled diagnosis in 6 (5.0%); and complete atrioventricular block in 2 (1.7%).

**Figure 1:**
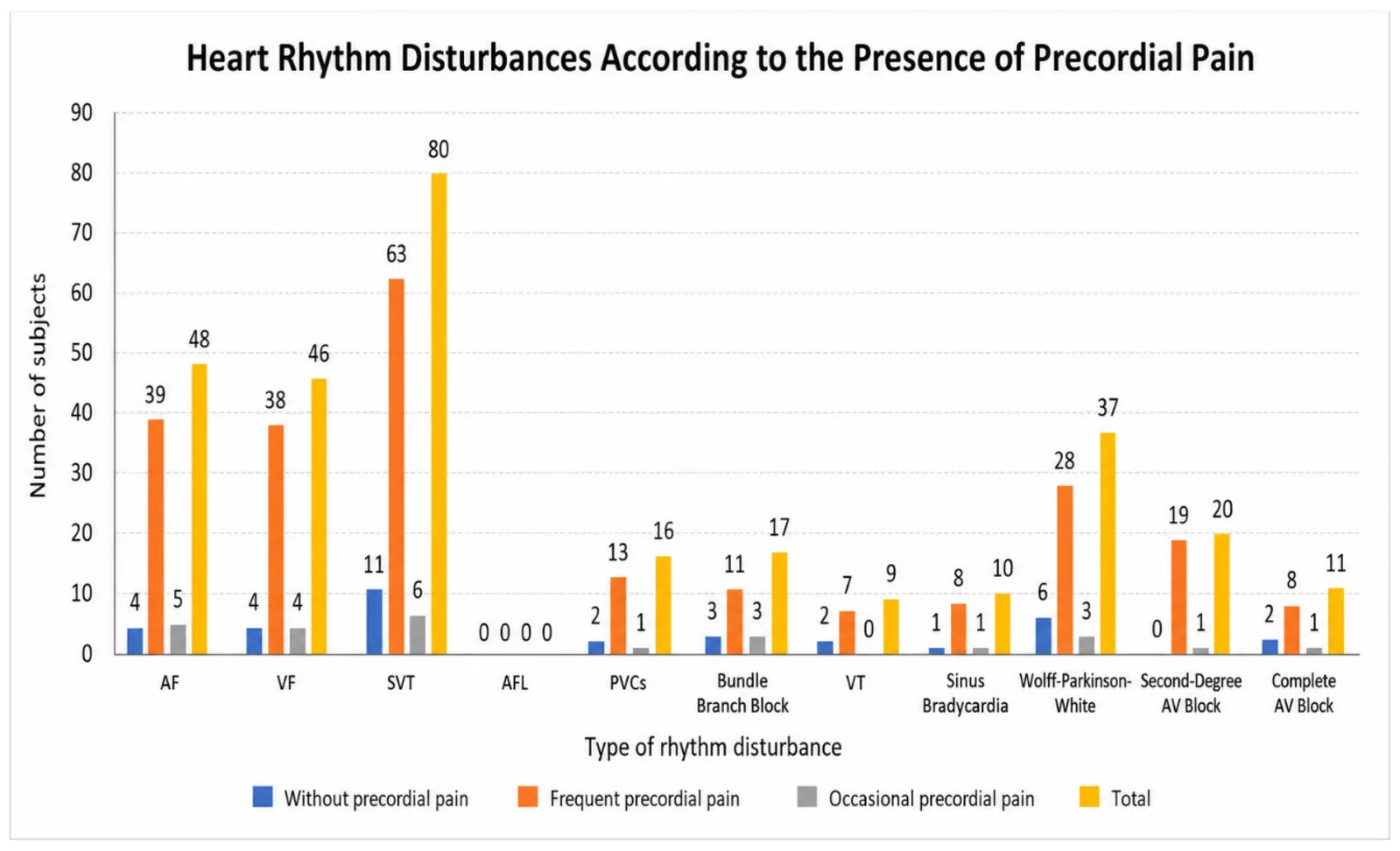
The frequency of cardiac rhythm disturbances according to the presence of precordial pain.

So, the symptom with the highest frequency for which these subjects presented to the hospital was the presence of precordial pain. A total of 105 subjects (88.23%) out of 119 reported having experienced episodes of precordial pain. Among these, 95 (79.83%) described the pain as persistent, while only 10 (8.40%) reported it as occasional (Figure 1).

### 3.4. Palpitations and Rhythm Findings

Palpitations are a fairly common reason for medical consultation in pediatric practice; however, a large proportion of these are not related to cardiac pathology. Describing the clinical context in which they occur, as well as their clinical characteristics, is extremely important in establishing the suspicion of a possible arrhythmia.

Palpitations were reported by 80 participants (67.2%), including 66 (55.5%) with frequent symptoms and 14 (11.8%) with occasional symptoms. Thirty-nine participants (32.8%) denied palpitations.

The relationship between palpitations and rhythm findings was evaluated.

The Spearman coefficients ranged from approximately 0.160 to 0.243 for several positive associations. These findings indicate statistical association in the analyzed dataset but do not establish clinically important explanatory power, temporal correlation, or causality. Because multiple rhythm outcomes were tested, the unadjusted *p*-values should not be interpreted independently as confirmatory evidence without multiplicity adjustment.

The reported analysis of palpitations and family history of sudden cardiac death did not demonstrate a statistically significant association between the two variables (Spearman’s ρ = −0.024, *p* = 0.739). The comparison of groups according to family history of sudden cardiac death also did not demonstrate a statistically significant difference in the reported test.

**Figure 2:**
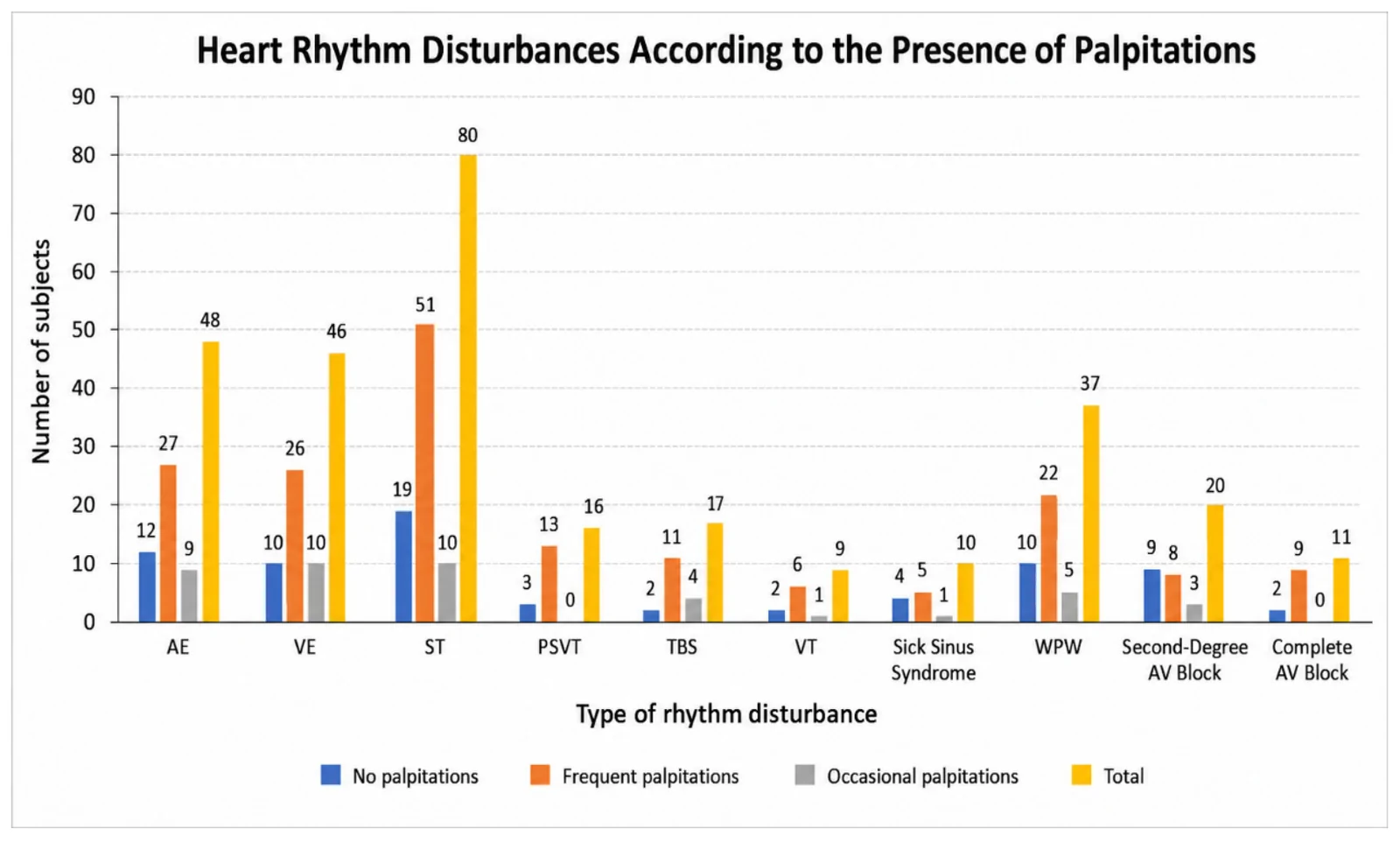
The frequency of cardiac rhythm disturbances according to the presence of palpitations.

As shown in Figure 2, we analyzed the association between palpitations and heart rhythm disturbances.

Given the well-known association between a family history of sudden death and the increased incidence of arrhythmias associated with a risk of sudden death, in our study we also analyzed—by performing the Spearman correlation tests and the *t*-test—whether there were statistically significant correlations between the presence of palpitations and a family history of sudden death.

Table 2 presents the results of Levene’s test for equality of variances and the independent-samples *t*-test. Levene’s test yielded a significance value of 0.736, which is greater than the conventional significance threshold of 0.05. Therefore, the assumption of equal variances is not violated, and the “Equal variances assumed” row is used for interpretation. The corresponding two-tailed significance value for the *t*-test was 0.867. Since this value is greater than 0.05, the null hypothesis cannot be rejected. Thus, the analysis did not identify a statistically significant difference between patients with and without a family history of sudden death.

As shown in Table 3, Spearman’s correlation analysis identified statistically significant associations between the presence of palpitations and several cardiac rhythm disorders. Significant correlations were observed with ventricular extrasystoles (VE) (ρ = 0.179, *p* = 0.012), systematized ventricular ectopy (ρ = 0.160, *p* = 0.024), sinus tachycardia (ρ = 0.160, *p* = 0.024), ventricular tachycardia (ρ = 0.168, *p* = 0.017), and complete atrioventricular (AV) block (ρ = −0.152, *p* = 0.032). Highly statistically significant correlations were observed for palpitations vs. tachy–brady syndrome (ρ = 0.196, *p* = 0.006) and palpitations vs. Wolff–Parkinson–White (WPW) syndrome (ρ = 0.243, *p* = 0.001).

Furthermore, a statistically significant positive correlation was identified between the presence of sudden death and complete AV block (ρ = 0.162, *p* = 0.023). No statistically significant correlations were observed between the presence of sudden death and the other cardiac rhythm disorders presented in the table (*p* > 0.05).

### 3.5. Syncope or Lipothymia

Syncope or lipothymia was reported by 39/119 participants (32.8%). The number of reported episodes was not normally distributed. Most participants reported a single episode, while fewer participants reported recurrent episodes. The reported circumstances of syncope included episodes associated with emotional or presumed vasovagal mechanisms and episodes occurring during or after physical exertion. These categories were based on the available clinical history and should not be interpreted as definitive etiological diagnoses.

Syncope is characterized by a transient reduction in cerebral perfusion sufficient to cause temporary loss of consciousness and postural tone. The available data do not support the assertion that exertional syncope results from excessive cerebral oxygen consumption followed by a protective fainting response [4].

Among the 39 participants with syncope or lipothymia, our data revealed the following rhythm findings: atrial extrasystoles in 13 (33.3%), including systematized atrial extrasystoles in 7 (17.9%); ventricular extrasystoles in 13 (33.3%), including systematized ventricular extrasystoles in 9 (23.1%); PSVT in 4 (10.3%); a WPW-labelled diagnosis in 7 (17.9%); tachy–brady syndrome in 5 (12.8%); sinus node dysfunction in 5 (12.8%); ventricular tachycardia in 3 (7.7%); and sinus tachycardia in 20 (51.3%).

The presence of syncope or lipothymia was evaluated in relation to selected rhythm diagnoses. The reported association with complete atrioventricular block was strong. However, because symptom timing during ambulatory monitoring was not recorded, this association should be interpreted as cross-sectional and cannot establish that complete atrioventricular block caused the reported syncopal episodes.

### 3.6. Symptom–Rhythm Analyses

For syncope/lipothymia and complete atrioventricular block, 10/39 participants with syncope/lipothymia had complete atrioventricular block compared with 1/80 participants without syncope/lipothymia. This corresponded to an odds ratio of 27.24 (95% CI, 3.34–222.29; Fisher’s exact *p* < 0.001). After Holm’s adjustment for the prespecified family of eight symptom–rhythm comparisons, the association is statistically significant (*p* = 0.0004).

The unadjusted association between syncope/lipothymia and the WPW-labelled diagnosis is not statistically significant after Holm’s adjustment. No statistically significant adjusted associations were identified between syncope/lipothymia and PSVT or ventricular tachycardia.

These results indicate cross-sectional association only. They do not establish that syncope was caused by the detected rhythm abnormality, nor do they demonstrate that syncope is a prognostic factor for future development of complete atrioventricular block.

### 3.7. Blood Pressure and Reported Syncope Episodes

The mean blood pressure value recorded in the cohort was 111.95 mmHg (SD, 17.311). The reported linear regression model relating blood pressure to the number of reported syncopal episodes yielded an R^2^ of 0.010.

An R^2^ value of 0.010 indicates that approximately 1% of the variance in the modeled outcome was explained by the predictor in that model. This represents negligible explanatory power and should not be described as a high coefficient of determination or as evidence of a clinically meaningful linear relationship.

### 3.8. Family History of Sudden Cardiac Death and Rhythm Findings

A family history of sudden cardiac death in a first- or second-degree relative before the age of 50 years was reported in 28/119 participants (23.5%).

Among the 39 participants with syncope or lipothymia, 12 (30.8%) also reported a family history of sudden cardiac death. This represented 10.1% of the entire cohort.

The coexistence of syncope and a family history of sudden cardiac death may identify a subgroup warranting careful clinical evaluation. However, the available cross-sectional data do not establish that this combination constitutes a validated risk factor for the presence of a specific rhythm disorder or for future sudden cardiac death.

Spearman’s correlation analysis was performed to investigate the associations between the presence of syncopal episodes (lipothymia) and selected cardiac rhythm disorders potentially associated with an increased risk of sudden cardiac death (Table 4).

Following the analysis, a statistically significant weak negative correlation was identified between the presence of syncopal episodes (lipothymia) and WPW syndrome (Spearman’s ρ = −0.198, *p* = 0.031). In addition, a statistically significant moderate positive correlation was observed between syncopal episodes and complete atrioventricular (AV) block (Spearman’s ρ = 0.395, *p* < 0.001). Therefore, the results indicate statistically significant associations between lipothymia and both WPW syndrome and complete AV block.

**Figure 3:**
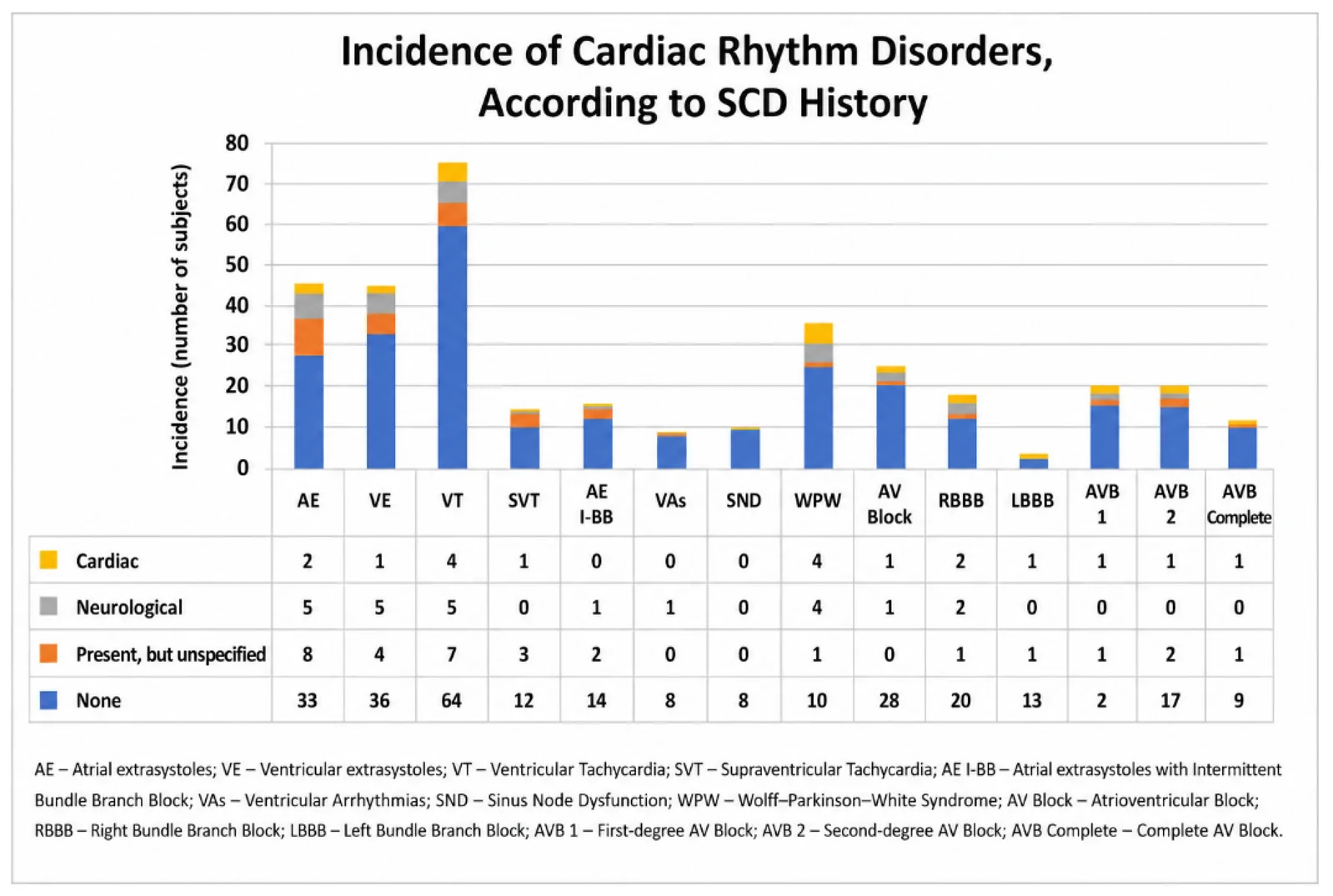
Frequency of cardiac rhythm disorders according to family history of sudden cardiac death.

As shown in Figure 3, we aimed to identify correlations between a history of sudden cardiac death and the overall frequency of cardiac arrhythmias in the entire group.

Based on the results presented in Table 5, statistically significant correlations were identified between the presence of sudden death and the diagnoses of WPW, sinus node dysfunction (SND), and complete atrioventricular (AV) block. Specifically, the presence of sudden death was weakly negatively correlated with WPW (Spearman’s ρ = −0.201, *p* = 0.028), and weakly positively correlated with complete AV block (ρ = 0.233, *p* = 0.011) and SND (ρ = 0.189, *p* = 0.040). All three associations reached statistical significance at the *p* < 0.05 level.

### 3.9. Descriptive Clinical Reassessment

Some participants attended routine clinical reassessment after their initial evaluation according to standard clinical practice. These reassessments were not prospectively scheduled as part of the study protocol, and no uniform follow-up duration or predefined assessment intervals were specified. Therefore, the information presented below is descriptive only. Among the 37 participants reported as having a WPW-labelled diagnosis, 33 attended individualized reassessment. Among the 11 participants reported as having complete atrioventricular block, 10 attended scheduled follow-up evaluations, including the two participants who underwent pacemaker implantation. Among the 16 participants reported as having PSVT, 13 attended scheduled reassessment.

### 3.10. Holter Monitoring and Management Impact

Of the 119 participants, 42 (35.3%) had a normal resting ECG, while 77 (64.7%) underwent ambulatory Holter monitoring.

Among the 77 patients undergoing Holter monitoring, rhythm abnormalities not seen on the resting ECG were detected in 65 patients. Holter findings resulted in at least one documented change in clinical management in 38/77 participants (49.4%) (Table 6). Management categories were not mutually exclusive because individual participants could experience more than one type of intervention.

The documented management changes included lifestyle or activity adjustment, including activity restrictions where clinically indicated, in 21 participants; initiation of antiarrhythmic therapy in 19; medication monitoring in 19; and targeted imaging, including cardiac magnetic resonance imaging, in 5.

The management categories therefore sum to more than 38 because some participants experienced more than one management change.

**Table 6:** Holter monitoring and management impact.

Finding or Management Outcome	*n*	%
Total study cohort	119	100.0
Normal resting ECG	42	35.3 *
Underwent Holter monitoring	77	64.7 *
Abnormality detected by Holter	65	84.4 ^†^
Any management change after Holter findings	38	49.4 ^†^
Lifestyle/activity adjustment	21	27.3 ^†^
Antiarrhythmic therapy initiated	19	24.7 ^†^
Medication monitoring	19	24.7 ^†^
Targeted imaging, including cardiac MRI	5	6.5 ^†^

* Percentages calculated using the total cohort (*n* = 119). ^†^ Percentages calculated using the Holter-monitored subgroup (*n* = 77). Management categories were not mutually exclusive.

### 3.11. Study-Derived Diagnostic Pathway

**Figure 4:**
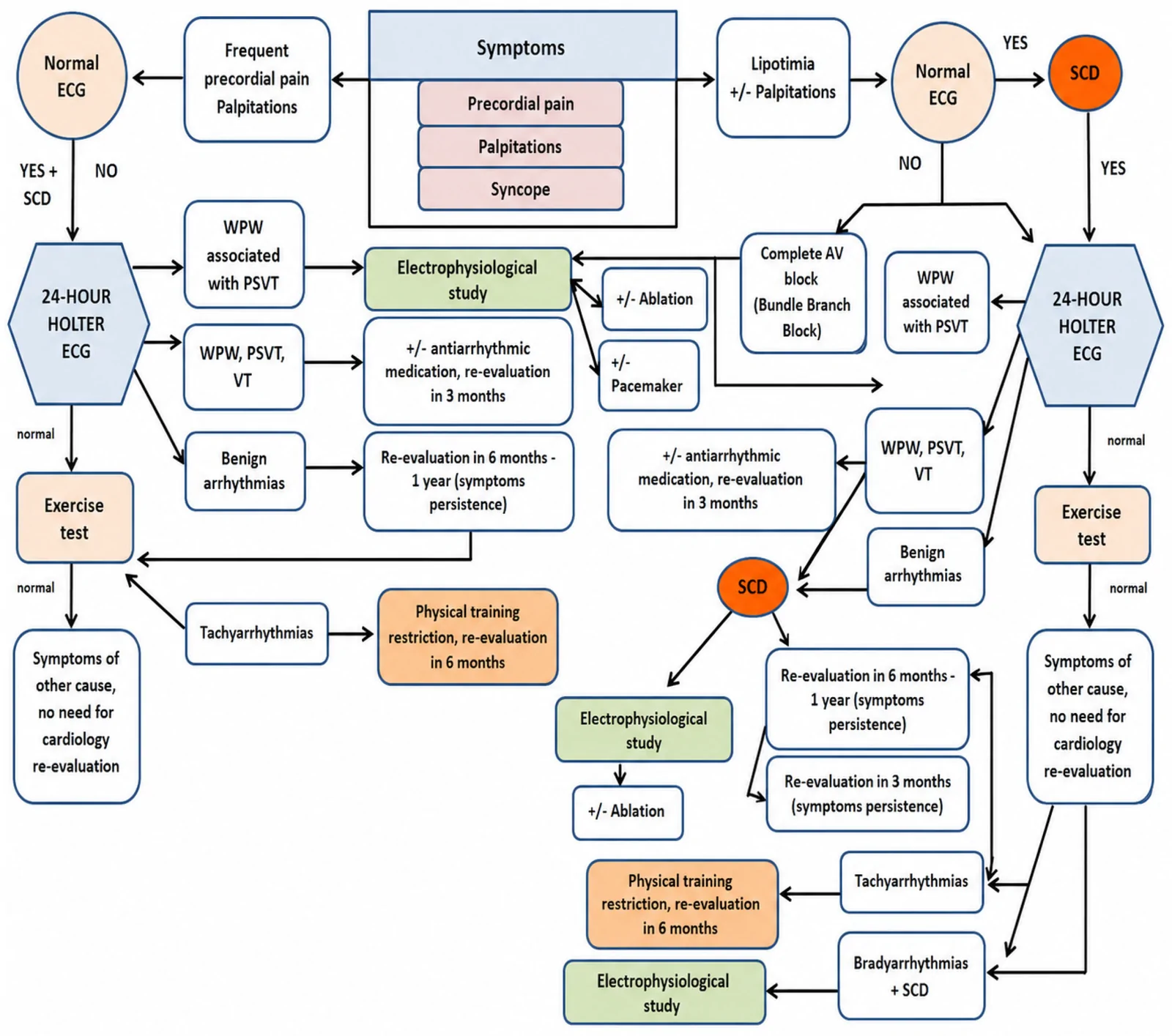
Protocol for the identification, evaluation, and follow-up monitoring of arrhythmias associated with a risk of sudden cardiac death in symptomatic children and adolescents (source: developed by the authors).

Considering that this is the first study published in Romania regarding the incidence of cardiac rhythm disorders in symptomatic children and adolescents, we developed a protocol (based on the clinical findings observed in this cohort) for the identification, evaluation, and follow-up monitoring of arrhythmias associated with a risk of sudden cardiac death in symptomatic children and adolescents (Figure 4). The proposed algorithm should be regarded as a study-derived clinical proposal rather than a formally validated diagnostic guideline. The specific follow-up intervals, treatment thresholds, and management pathways shown in the algorithm reflect the findings of the present cohort and expert interpretation of these findings and should therefore be adapted to the individual clinical context and local clinical practice. The pathway should not be interpreted as prospectively validated.

## 4. Discussion

Cardiac rhythm disorders in children and adolescents represent an important diagnostic challenge because their clinical presentation may be variable, intermittent, and frequently nonspecific. Although some studies have reported a higher incidence of rhythm disorders among males, with ratios as high as 9:1 [9,10,11,12], other investigations have described a more balanced distribution between the sexes [13]. In the present cohort, females represented 63.0% of the participants, compared with 37.0% males. This distribution should be interpreted as a characteristic of the study population and should not be considered evidence that cardiac rhythm disorders are generally more prevalent among females.

Several factors may contribute to the development of rhythm disturbances during childhood and adolescence. Lower respiratory tract infections, particularly those caused by respiratory syncytial virus, may occasionally be associated with cardiac rhythm disturbances during the acute phase [14]. In addition, viral myocarditis may occur as a complication of infectious disease and may manifest with arrhythmias. Myocardial inflammation may also result in structural lesions that persist after the acute infection and potentially provide a substrate for subsequent rhythm disorders [15,16,17].

Based on these considerations, we investigated whether a history of lower respiratory tract disease was associated with the rhythm disorders identified in our cohort. The analysis did not identify statistically significant associations between a history of lower respiratory tract disease and the rhythm disorders evaluated. The observed frequency of rhythm disorders was generally higher among participants without a documented history of pneumonia. These findings should be interpreted cautiously, particularly because the study was cross-sectional and the absence of a documented history of pneumonia does not exclude previous infection or other potentially relevant inflammatory or infectious exposures.

The clinical manifestations of cardiac rhythm disorders in children and adolescents may be highly variable [18]. The literature indicates that a considerable proportion of arrhythmias may be asymptomatic, with symptoms becoming more apparent with increasing age [19,20]. In the present study, all participants were evaluated because of symptoms that could potentially be associated with cardiac rhythm disorders. The most frequently reported presenting symptom was precordial pain, reported by 105 of 119 participants (88.2%), followed by palpitations, reported by 80 participants (67.2%). Lipothymia or syncope was reported by 39 participants (32.8%), while vertigo and dyspnea were also frequently documented.

Precordial pain is a common reason for pediatric medical consultation and is often a source of considerable concern for caregivers. In contrast to adults, however, the majority of pediatric cases are not caused by cardiac disease [21,22,23,24,25]. Nevertheless, a small proportion of children presenting with precordial pain may have an underlying cardiac disorder, including a rhythm disturbance. Previous studies have emphasized the importance of considering paroxysmal supraventricular tachycardia, ventricular tachycardia, and bradyarrhythmias in the differential diagnosis of children with potentially cardiac precordial pain [26].

In our cohort, the frequency of rhythm diagnoses varied according to the reported presence and characteristics of precordial pain. However, because the study did not record whether the symptoms occurred simultaneously with a documented rhythm abnormality, these findings should be interpreted as cross-sectional associations rather than as evidence of a temporal relationship between precordial pain and a specific arrhythmia. Therefore, the presence of precordial pain alone cannot be considered a direct predictor of a particular rhythm disorder. Nevertheless, recurrent or unexplained precordial pain remains a clinically relevant reason for further cardiovascular evaluation, particularly when accompanied by other symptoms such as palpitations, syncope, exertional symptoms, or a relevant family history.

Palpitations were reported by 80 participants (67.2%), including 66 participants (55.5%) with frequent symptoms and 14 (11.8%) with occasional symptoms. Thirty-nine participants (32.8%) reported no palpitations. Sudden-onset palpitations with abrupt termination, particularly in the absence of fever or other acute illness, may raise suspicion of a cardiac rhythm disorder [27,28,29]. Palpitations may occur in association with atrial or ventricular ectopy, ventricular tachycardia, or pre-excitation syndromes [19]. Although most palpitations in children and adolescents are benign, a cardiac cause should be considered when symptoms are recurrent, abrupt in onset, associated with syncope or exertion, or accompanied by an abnormal electrocardiographic finding. The clinical assessment should include a detailed history, physical examination, and electrocardiography, with Holter monitoring, exercise testing, and echocardiography considered when clinically indicated [30].

In the present study, Spearman’s correlation analysis identified statistically significant associations between palpitations and several documented rhythm diagnoses. These included ventricular extrasystoles, systematized ventricular ectopy, sinus tachycardia, ventricular tachycardia, complete atrioventricular block, tachy–brady syndrome, and WPW. The correlation coefficients were generally weak, ranging from approximately 0.160 to 0.243 in the positive associations, while the correlation between palpitations and complete AV block was weak and negative (ρ = −0.152, *p* = 0.032). The strongest associations were observed for WPW (ρ = 0.243, *p* = 0.001) and tachy–brady syndrome (ρ = 0.196, *p* = 0.006).

These findings indicate statistical associations within the study population but do not establish that palpitations were caused by the documented rhythm abnormalities. Furthermore, because multiple rhythm outcomes were evaluated, the unadjusted *p*-values should be interpreted with caution, as multiple testing increases the possibility of chance findings. The association between palpitations and a history of sudden cardiac death was not statistically significant (ρ = −0.024, *p* = 0.739). Similarly, the independent-samples comparison did not demonstrate a statistically significant difference between the groups in the reported analysis (*p* = 0.867). Thus, the data do not support the conclusion that palpitations were significantly associated with a family history of sudden cardiac death in this cohort.

Syncope or lipothymia was reported by 39 of the 119 participants (32.8%). Syncope in children and adolescents is relatively common and may have a variety of causes, including vasovagal mechanisms, orthostatic mechanisms, and cardiac disorders. Exertional syncope is of particular clinical concern because it may occur in association with an underlying cardiac disorder. Previous authors have emphasized the importance of electrocardiographic evaluation and, when clinically indicated, ambulatory monitoring in the assessment of patients with unexplained or exertional syncope [31,32].

Most syncopal episodes in the pediatric population are benign and vasovagal in nature, although a proportion may be associated with cardiac disease or rhythm disturbances [32,33,34]. Previous studies have identified rhythm disorders such as WPW, complete AV block, sinus node dysfunction, and PSVT among children evaluated for syncope [35]. In the present cohort, the rhythm findings among participants with lipothymia included atrial extrasystoles, ventricular extrasystoles, PSVT, a WPW-labelled diagnosis, tachy–brady syndrome, sinus node dysfunction, ventricular tachycardia, and sinus tachycardia.

The most notable association was observed between lipothymia and complete AV block. Ten of the 39 participants with lipothymia had complete AV block, compared with one of the 80 participants without lipothymia. This corresponded to an odds ratio of 27.24 (95% CI, 3.34–222.29), with Fisher’s exact *p* < 0.001. The association remained statistically significant after Holm’s adjustment for the prespecified family of symptom–rhythm comparisons (adjusted *p* = 0.0004). This finding indicates a strong cross-sectional association between lipothymia and complete AV block in this cohort.

However, this association should not be interpreted as proof that complete AV block caused the reported syncopal episodes. The timing of symptoms in relation to rhythm events was not systematically documented, and the cross-sectional study design does not establish temporal sequence or causality. Similarly, the association does not demonstrate that syncope is a prognostic factor for the future development of complete AV block. Nevertheless, the finding emphasizes the importance of careful rhythm evaluation in children and adolescents presenting with unexplained syncope, particularly when other clinical or electrocardiographic features raise suspicion of an underlying rhythm disorder.

The relationship between syncope and WPW requires a similarly cautious interpretation. The Spearman analysis identified a weak negative correlation between lipothymia and WPW (ρ = −0.198, *p* = 0.031). However, the more detailed symptom–rhythm analysis indicated that the unadjusted association did not remain statistically significant after Holm’s adjustment. This illustrates the importance of accounting for multiple comparisons when interpreting several simultaneous statistical tests. Accordingly, the present findings do not provide robust evidence of an independent association between syncope and WPW after adjustment for multiple testing.

The relationship between blood pressure and the number of reported syncopal episodes was also evaluated. The regression model yielded an R^2^ of 0.010, indicating that approximately 1% of the variance in the modeled outcome was explained by the predictor. This represents negligible explanatory power. Therefore, the results do not support a clinically meaningful linear relationship between the blood pressure measurement evaluated and the number of reported syncopal episodes in this cohort.

A family history of sudden cardiac death represents an important clinical feature that may raise suspicion of inherited arrhythmic disorders or cardiomyopathies [36,37,38,39,40,41]. Accordingly, a detailed family history is an important component of the assessment of children and adolescents presenting with syncope, palpitations, or other potentially cardiac symptoms [42,43,44]. In our cohort, 28 of 119 participants (23.5%) reported a first- or second-degree relative who had died suddenly before the age of 50 years. Among the 39 participants with lipothymia, 12 (30.8%) also reported a family history of sudden cardiac death, corresponding to 10.1% of the total cohort.

The coexistence of syncope and a family history of sudden cardiac death identifies a clinically relevant subgroup that may warrant particularly careful cardiovascular evaluation. However, the present cross-sectional data do not establish that this combination constitutes a validated independent risk factor for a specific rhythm disorder or for future sudden cardiac death. In addition, the presence of a family history does not necessarily indicate that an individual patient has an inherited arrhythmic disorder. Rather, it should prompt careful clinical assessment, detailed evaluation, electrocardiographic examination, and further investigation when clinically indicated [42,43,44].

In the present study, statistically significant correlations were identified between the reported presence of sudden cardiac death in the family and WPW, complete AV block, and sinus node dysfunction. The correlations were weak: WPW was negatively correlated with the reported presence of sudden cardiac death (ρ = −0.201, *p* = 0.028), whereas complete AV block (ρ = 0.233, *p* = 0.011) and sinus node dysfunction (ρ = 0.189, *p* = 0.040) showed weak positive correlations. These findings demonstrate statistical associations within this sample but should not be interpreted as evidence that a family history of sudden cardiac death causes these rhythm disorders or independently predicts their development. The relatively modest correlation coefficients also indicate that the observed relationships explain only a limited proportion of the variability between the variables.

The relationship between family history and WPW is of particular clinical interest because the previous literature has suggested a possible familial component to pre-excitation syndromes, although familial cases are uncommon [45]. In our cohort, none of the participants with a WPW-labelled diagnosis had a known first-degree relative with the same condition. This finding does not exclude a genetic contribution, as the study did not include systematic genetic testing or comprehensive screening of relatives. It also illustrates the difference between a family history of sudden cardiac death and a family history of WPW itself. These should be considered separate clinical variables.

The results also provide important information regarding the diagnostic value of ambulatory monitoring. In the present cohort, 77 of 119 participants (64.7%) underwent Holter monitoring. Among these participants, 65 (84.4%) had rhythm abnormalities detected exclusively by ambulatory monitoring and not identified on the resting electrocardiogram. This finding emphasizes the limitations of a single resting ECG in patients with intermittent rhythm disturbances. It also supports the clinical value of prolonged rhythm monitoring in selected symptomatic children and adolescents, particularly those with recurrent palpitations, unexplained syncope, exertional symptoms, or other clinical features suggesting an intermittent arrhythmia.

The diagnostic findings from Holter monitoring also had a direct impact on clinical management. A documented management change occurred in 38 of the 77 participants who underwent Holter monitoring (49.4%). These changes included lifestyle or activity adjustment, initiation of antiarrhythmic therapy, medication monitoring, and targeted imaging, including cardiac magnetic resonance imaging. Because some participants experienced more than one type of management change, these categories were not mutually exclusive. The results therefore suggest that ambulatory monitoring was not only diagnostically useful but also clinically relevant in influencing subsequent management decisions.

Overall, the present study highlights the complexity of evaluating symptoms that may be associated with cardiac rhythm disorders in children and adolescents. Precordial pain and palpitations were common presenting symptoms, but their presence alone did not establish the existence of a specific rhythm disorder. The most clinically important association identified in the present cohort was between lipothymia and complete AV block, although the cross-sectional design prevents conclusions regarding causality. A family history of sudden cardiac death was relatively common in the study population and was associated with weak correlations with WPW, complete AV block, and sinus node dysfunction, but these findings should be regarded as exploratory and require confirmation in larger prospective cohorts.

The high diagnostic yield of ambulatory monitoring and its effect on clinical management represent important practical findings. In symptomatic children and adolescents, particularly those with intermittent symptoms that may not be captured during a standard ECG examination, prolonged monitoring may substantially improve the detection of clinically relevant rhythm abnormalities. Nevertheless, the results should be interpreted in light of the study’s limitations, including its cross-sectional design, the absence of systematic documentation linking symptoms temporally to rhythm events, the relatively limited sample size, and the potential for chance findings resulting from multiple statistical comparisons. Future prospective studies involving larger cohorts, standardized symptom–rhythm event recording, systematic family evaluation, and, where appropriate, genetic assessment are needed to further clarify the relationships between symptoms, family history, rhythm disorders, and the risk of sudden cardiac death.

## 5. Conclusions

The study cohort was predominantly composed of participants from urban areas and included a higher proportion of females. The sex distribution observed in this cohort differs from that reported in some previous studies and should be interpreted as a characteristic of the present study population rather than as evidence of a generally higher incidence of cardiac rhythm disorders among females.

The most frequently reported symptom was precordial pain, followed by palpitations and lipothymic or syncopal episodes. The presence of these symptoms alone did not establish a specific rhythm diagnosis.

Statistically significant associations were identified between palpitations and several cardiac rhythm disorders, including ventricular extrasystoles, systematized ventricular ectopy, sinus tachycardia, ventricular tachycardia, complete atrioventricular block, tachy–brady syndrome, and WPW. The observed correlations were generally weak. The strongest associations were observed for palpitations vs. WPW and palpitations vs. tachy–brady syndrome. These findings demonstrate statistical associations but do not establish causality or prove that the documented rhythm disorders were responsible for the reported palpitations.

Approximately one-third of the study population reported lipothymic or syncopal episodes. The most clinically relevant association identified was between lipothymia and complete atrioventricular block. Participants with lipothymia were substantially more likely to have complete AV block than participants without lipothymia, and this association remained statistically significant after adjustment for multiple comparisons. An unadjusted statistically significant association was also observed between lipothymia and WPW; however, this association did not remain statistically significant after adjustment for multiple testing.

A family history of sudden cardiac death before the age of 50 years in a first- or second-degree relative was reported by approximately one-quarter of the study population. Among participants with lipothymia, approximately one-third also reported such a family history. Statistically significant but weak correlations were identified between the reported presence of sudden cardiac death and WPW, complete atrioventricular block, and sinus node dysfunction. These findings indicate associations within the study cohort but do not establish causality or demonstrate that family history independently predicts the development of a specific rhythm disorder or sudden cardiac death.

The findings indicate that the coexistence of lipothymia and a family history of sudden cardiac death represents a clinically relevant combination of findings that may warrant particularly careful cardiovascular assessment. However, the present study does not establish that this combination independently constitutes a validated risk factor for lethal arrhythmias or future sudden cardiac death.

Ambulatory Holter monitoring demonstrated substantial diagnostic value in the evaluated cohort. The majority of participants who underwent Holter monitoring had rhythm abnormalities detected exclusively through ambulatory monitoring rather than on the resting ECG, and approximately half had a documented change in clinical management following the monitoring results. These findings support the use of prolonged ambulatory rhythm monitoring in selected symptomatic children and adolescents, particularly when symptoms are intermittent or are not explained by a standard resting ECG.

Overall, the findings emphasize the importance of a comprehensive cardiovascular evaluation in symptomatic children and adolescents, particularly those presenting with unexplained lipothymia or syncope, recurrent palpitations, exertional symptoms, or a family history of sudden cardiac death. The assessment should integrate a detailed clinical-history–family-history evaluation, electrocardiography, and ambulatory rhythm monitoring when clinically indicated. The results should be interpreted in the context of the cross-sectional study design, and the observed associations should be further evaluated in larger prospective studies.

### 5.1. Future Research Perspectives

- Long-term follow-up over the next 10–15 years of subjects identified with arrhythmias associated with a risk of sudden death is needed to further stratify the incidence of sudden death within this population.
- A dedicated study on the Wolff–Parkinson–White syndrome within the same sociodemographic area should be conducted, with the aim of identifying the factors underlying the notably high incidence of this syndrome. Additionally, first-degree relatives of these subjects should be evaluated to establish potential correlations regarding the genetic component of the condition.

### 5.2. Study Limitations

This study has several limitations. First, its single-center design may limit the representativeness of the study population and introduce institution-specific referral and management patterns. Second, the relatively modest sample size limits the statistical precision of the observed estimates and reduces the ability to perform robust subgroup analyses, particularly across individual types of cardiac rhythm disorders. Therefore, the observed sex distribution and other associations should be interpreted cautiously.

Third, referral bias cannot be excluded. As the study was conducted in a specialized clinical setting, patients referred for evaluation may have had more severe, symptomatic, recurrent, or diagnostically challenging rhythm disorders than patients managed in primary care or general cardiology settings. Consequently, the distribution of arrhythmias observed in this cohort may not reflect their prevalence in the general population. Conversely, patients with less symptomatic or undiagnosed rhythm disorders may have been underrepresented.

Although follow-up attendance was available for some participants through routine clinical care, the study did not include a predefined longitudinal follow-up protocol with standardized timing or duration. Consequently, no longitudinal outcome analyses could be performed.

The generalizability of the findings may be limited by the demographic and clinical characteristics of the study population, the healthcare setting, and local diagnostic and referral practices. The observed female predominance in this cohort may therefore not be applicable to other populations or healthcare systems. Multicenter studies involving larger and more diverse populations, ideally with prospective follow-up and external validation of the proposed algorithm, are needed to confirm the observed patterns and determine whether the proposed diagnostic pathway improves diagnostic yield, risk stratification, or clinical outcomes.

## Figures and Tables

**Table 1:** Percentage distribution of presenting symptoms.

**Vertigo ^a^**					
		**Frequency**	**Percent**	**Valid Percent**	**Cumulative Percent**
Valid	No	94	79.0	79.0	79.0
	Yes	21	17.6	17.6	96.6
	Occasionally	4	3.4	3.4	100.0
	Total	119	100.0	100.0	
**Precordial pain ^a^**					
		**Frequency**	**Percent**	**Valid Percent**	**Cumulative Percent**
Valid	No	14	11.8	11.8	11.8
	Yes	95	79.8	79.8	91.6
	Occasionally	10	8.4	8.4	100.0
	Total	119	100.0	100.0	
**Palpitations ^a^**					
		**Frequency**	**Percent**	**Valid Percent**	**Cumulative Percent**
Valid	No	39	32.8	32.8	32.8
	Yes	66	55.5	55.5	88.2
	Occasionally	14	11.8	11.8	100.0
	Total	119	100.0	100.0	
**Dyspnea ^a^**					
		**Frequency**	**Percent**	**Valid Percent**	**Cumulative Percent**
Valid	No	50	42.0	42.0	42.0
	Yes	3	2.5	2.5	44.5
	Medium	4	3.4	3.4	47.9
	Moderated by effort	3	2.5	2.5	50.4
	Occasionally by effort	1	0.8	0.8	51.3
	Of effort	58	48.7	48.7	100.0
	Total	119	100.0	100.0	
**Lipothymia ^a^**					
		**Frequency**	**Percent**	**Valid Percent**	**Cumulative Percent**
Valid	No	80	67.2	67.2	67.2
	Yes	39	32.8	32.8	100.0
	Total	119	100.0	100.0	

^a^ Athlete = No.

**Table 2:** Are there significant differences among the subjects according to the history of sudden death?

		Levene’s Test for Equality of Variances		*t*-Test for Equality of Means		
		F	Sig.	t	df	Sig. (2-Tailed)
Presence of sudden death	Equal variances assumed	0.114	0.736	0.168	117	0.867
	Equal variances not assumed			0.169	171.164	0.866

**Table 3:** Spearman’s test regarding the correlations between palpitations, a history of sudden death, and cardiac rhythm disorders.

			Palpitations	Presence of Sudden Death
Spearman’s rho	Palpitations	Correlation Coefficient	1.000	−0.024
		Sig. (2-tailed)		0.739
		*n*	119	119
	Presence of sudden death	Correlation Coefficient	−0.024	1.000
		Sig. (2-tailed)	0.739	
		*n*	119	119
		Sig. (2-tailed)	0.053	0.943
		*n*	118	118
	VE	Correlation Coefficient	0.179	0.040
		Sig. (2-tailed)	0.012	0.576
		*n*	119	119
	Systematized_VE	Correlation Coefficient	0.160	−0.127
		Sig. (2-tailed)	0.024	0.073
		*n*	119	119
	ST	Correlation Coefficient	0.160	−0.127
		Sig. (2-tailed)	0.024	0.073
		*n*	119	119
	TACHY–BRADY	Correlation Coefficient	0.196	−0.106
		Sig. (2-tailed)	0.006	0.134
		*n*	119	119
	VT	Correlation Coefficient	0.168	−0.048
		Sig. (2-tailed)	0.017	0.496
		*n*	119	119
	WPW	Correlation Coefficient	0.243	−0.132
		Sig. (2-tailed)	0.001	0.063
		*n*	119	119
	Complete AV block	Correlation Coefficient	−0.152	0.162
		Sig. (2-tailed)	0.032	0.023
		*n*	119	119

**Table 4:** Spearman’s rho test regarding correlations between syncopal episodes (lipothymia) and arrhythmias with risk of sudden cardiac death.

			TPSV	TV	WPW	Complete AV Block	Lipothymia
Spearman’s rho	WPW	Correlation Coefficient	−0.212	0.014	1.000	−0.214	−0.198
		Sig. (2-tailed)	0.021	0.881		0.019	0.031
		*n*	119	119	119	119	119
	Complete AV block	Correlation Coefficient	−0.126	0.018	−0.214	1.000	0.395
		Sig. (2-tailed)	0.173	0.842	0.019		0.000
		*n*	119	119	119	119	119

**Table 5:** Correlations regarding the history of sudden cardiac death, syncopal episodes (lipothymia), and arrhythmias with risk of sudden cardiac death.

Correlations									
			TPSV	TV	WPW	Complete AV Block	SND	Presence of Sudden Death	Lipothymia
Spearman’s rho	WPW	Correlation Coefficient	−0.212 *	0.014	1.000	−0.214 *	−0.203 *	−0.201 *	−0.198 *
		Sig. (2-tailed)	0.021	0.881		0.019	0.026	0.028	0.031
		*n*	119	119	119	119	119	119	119
	Complete AV block	Correlation Coefficient	−0.126	0.018	−0.214 *	1.000	0.112	0.233 *	0.395 **
		Sig. (2-tailed)	0.173	0.842	0.019		0.223	0.011	0.000
		*n*	119	119	119	119	119	119	119
	SND	Correlation Coefficient	−0.119	0.028	−0.203 *	0.112	1.000	0.189 *	0.111
		Sig. (2-tailed)	0.196	0.763	0.026	0.223		0.040	0.229
		*n*	119	119	119	119	119	119	119

* Correlation is significant at the 0.05 level (2-tailed). ** Correlation is significant at the 0.01 level (2-tailed).

## Data Availability

The original contributions presented in this study are included in the article. Further inquiries can be directed to the corresponding author.
